# Repurposing FDA-Approved Agents to Develop a Prototype *Helicobacter pylori* Shikimate Kinase (HPSK) Inhibitor: A Computational Approach Using Virtual Screening, MM-GBSA Calculations, MD Simulations, and DFT Analysis

**DOI:** 10.3390/ph18020174

**Published:** 2025-01-27

**Authors:** Abdulaziz H. Al Khzem, Tagyedeen H. Shoaib, Rua M. Mukhtar, Mansour S. Alturki, Mohamed S. Gomaa, Dania Hussein, Nada Tawfeeq, Mohsina Bano, Mohammad Sarafroz, Raghad Alzahrani, Hanin Alghamdi, Thankhoe A. Rants’o

**Affiliations:** 1 Department of Pharmaceutical Chemistry, College of Clinical Pharmacy, Imam Abdulrahman Bin Faisal University, P.O. Box 1982, Dammam 31441, Saudi Arabia; msmmansour@iau.edu.sa (M.S.G.); nztawfeeq@iau.edu.sa (N.T.); mbshaik@iau.edu.sa (M.B.); mskausar@iau.edu.sa (M.S.); 2 Department of Pharmaceutical Chemistry, Faculty of Pharmacy, University of Gezira, Wad Madani 21111, Sudan; shoaibth37@hotmail.com (T.H.S.); ruamubarak1@gmail.com (R.M.M.); 3 Department of Pharmacology, College of Clinical Pharmacy, Imam Abdulrahman Bin Faisal University, Khobar 31441, Saudi Arabia; dahussein@iau.edu.sa; 4 College of Clinical Pharmacy, Imam Abdulrahman Bin Faisal University, P.O. Box 1982, Dammam 31441, Saudi Arabia; 2200004027@iau.edu.sa (R.A.); 2200005916@iau.edu.sa (H.A.); 5 Department of Pharmacology and Toxicology, College of Pharmacy, University of Utah, Salt Lake City, UT 84112, USA; thankhoe.rantso@pharm.utah.edu

**Keywords:** *Helicobacter pylori*, shikimate kinase, repurposing, computational studies, molecular simulations, DFT analysis

## Abstract

**Background/Objectives:** *Helicobacter pylori* infects approximately half of the global population, causing chronic gastritis, peptic ulcers, and gastric cancer, a leading cause of cancer mortality. While current therapies face challenges from rising antibiotic resistance, particularly to clarithromycin, alongside treatment complexity and costs, the World Health Organization has prioritized the development of new antibiotics to combat this high-risk pathogen. In this study, we employed computer-aided drug design (CADD) methodologies, including molecular docking, Molecular Mechanics-Generalized Born Surface Area (MM-GBSA) analysis, molecular dynamics (MD) simulations, and Density Functional Theory (DFT) calculations, to explore the potential repurposing of FDA-approved agents as inhibitors of *Helicobacter pylori* shikimate kinase (HpSK). **Methods:** Using the Glide module, the HTVS method was initially applied to screen 1615 FDA-approved agents followed by extra-precision (XP) docking for the obtained 111 hits. The obtained XP scores were used to confine the results to those hits with a score above the reference ligand, shikimate, score. This yielded 31 final hits with an XP score above −5.867. MM-GBSA calculations were performed on these top candidates and the reference ligand to refine the analysis and compounds’ prioritization. **Results:** The 31 compounds displayed binding free energy (ΔG_bind_) values ranging from 3.61 to −55.92 kcal/mol, with shikimate exhibiting a ΔG_bind_ of −34.24 kcal/mol and 10 hits having a lower ΔG_bind_ value. Out of these ten, three drugs—Dolutegravir, Cangrelor, and Isavuconazonium—were selected for further analysis based on their drug-like properties. Robust and stable binding profiles for both Isavuconazonium and Cangrelor were verified via molecular dynamics simulation. Additionally, Density Functional Theory (DFT) analysis was conducted, and the Highest Occupied Molecular Orbitals (HOMOs), Lowest Unoccupied Molecular Orbitals (LUMOs), and the energy gap (HLG) between them were calculated. All three drug candidates displayed lower HLG values than shikimate, suggesting higher reactivity and more efficient electronic transitions than the reference ligand. **Conclusions:** These findings suggest that the identified drugs, although not optimal for direct repurposing, would serve as promising leads against *Helicobacter pylori* shikimate kinase. These drugs could be valuable leads for experimental assessment and further optimization, particularly with no prototype yet identified. In terms of potential for clinical repurposing, the results point to diflunisal as a promising candidate for further testing.

## 1. Introduction

*Helicobacter pylori* (*H. pylori*), a Gram-negative spiral-shaped bacterium, colonizes the human stomach, leading to chronic infection [1,2]. It is estimated that 50% of the world’s population is infected by *H. pylori*. Although 80–90% of infected individuals show no symptoms, chronic infection can result in gastritis and peptic ulcers, or advance to cause gastric cancers [3]. Since 1994, the International Agency for Research on Cancer (IARC) has classified *H. pylori* as a Group 1 carcinogen, owing to its definitively established gastric carcinogenesis, as evidenced by epidemiologic studies [4]. Gastric cancer is a highly lethal malignant tumor that begins at the stomach mucosa level, disseminates to the other layers of the stomach wall, and evades adjacent and distant body parts in the most advanced stages [5]. In 2020, gastric cancer was the fifth most common cancer worldwide, with approximately 1.1 million new cases, and the fourth leading cause of cancer-related deaths, with around 800,000 deaths [6].

For more than two decades, *H. pylori* has been treated using triple therapy. In the first line, the therapy includes a proton pump inhibitor coupled with amoxicillin and clarithromycin. However, metronidazole-containing triple therapy or bismuth-containing quadruple therapy are used when clarithromycin resistance is present [7,8]. Currently, the major obstacle to eradicating *H. pylori* is the emergence of drug resistance, which deteriorates the efficacy of eradication regimens [9]. According to the World Health Organization (WHO), clarithromycin-resistant *H. pylori* is listed as a high-priority target bacterium for the discovery, research, and development of new antibiotics [10]. Additionally, other factors such as treatment complexity, side effects, and costs necessitate the discovery of new antibiotics to combat *H. pylori* [9].

**Figure 1:**
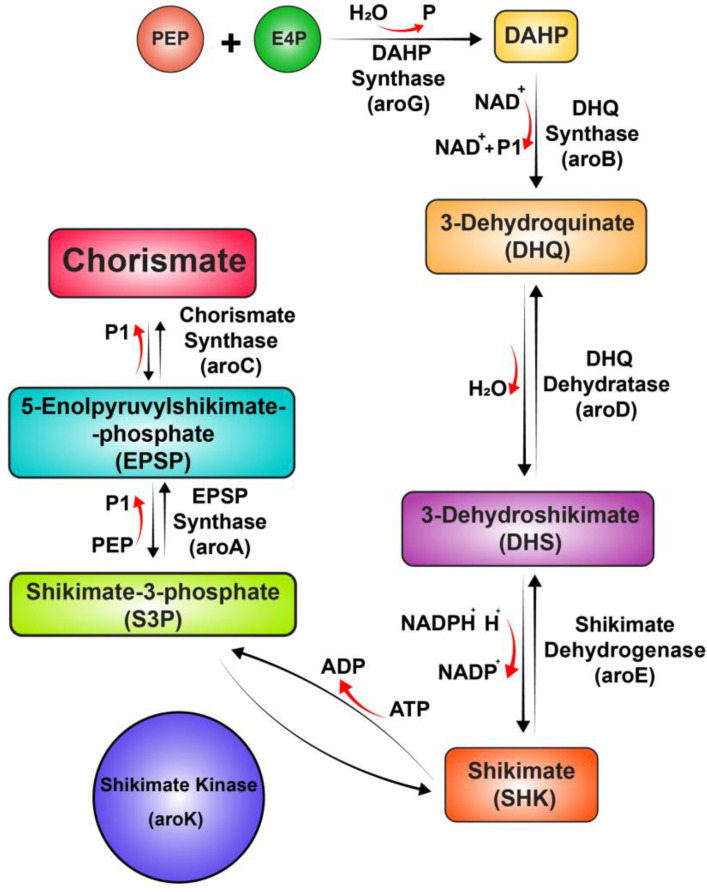
The shikimate pathway (SP).

Advancing research and developing new antibiotics with unique mechanisms of action that provide high-efficacy and low-toxicity profiles are crucial for reducing the risk of drug resistance and expanding the antimicrobial arsenal [11,12]. In this context, the shikimate pathway (SP) represents a goldmine for finding new antimicrobials. The SP is a central metabolic pathway consisting of seven metabolic steps catalyzed by seven enzymes: 3-deoxy-D-arabino-heptulosonate 7-phosphate synthase (DAHPS), 3-dehydroquinate synthase (DHQS), DHQ dehydratase (DHQD), shikimate dehydrogenase (SDH), 5-enolpyruvylshikimate 3-phosphate synthase (EPSPS), and chorismate synthase (CS). Following the sequence of the aforementioned enzymes results in the transformation of phosphoenolpyruvate and erythrose 4-phosphate to chorismate—a precursor of essential metabolites such as aromatic amino acids, folic acid, and ubiquinone (Figure 1) [13,14]. The SP is present in microbes and plants but absent in animals, making it a promising target for the discovery of selective antimicrobials and herbicides [15]. Extensive efforts have been dedicated towards uncovering compounds that interfere with the SP. Various natural, semi-synthetic, and synthetic inhibitors targeting distinct enzymes within the pathway have been identified. However, none of these inhibitors have successfully obtained approval as antimicrobials or herbicides, with the exception of glyphosate, a well-known herbicide that exerts its effect by inhibiting EPSPS [11].

As an intriguing drug target that meets the criteria for novelty and selectivity to address resistance, there remains a notable gap in the discovery of approved drugs that specifically target it. Intending to participate in filling this gap, in the current study, we targeted one of the key enzymes in the SP: shikimate kinase (SK), which catalyzes the phosphorylation of shikimate to form shikimate 3-phosphate in the fifth step of the metabolic sequence [16]. SK is classified as a P-loop kinase, sharing a common alpha–beta–alpha fold structure with conserved residues that create its active site, typically occupied by ATP and shikimate [15]. When the gene encoding SK (aroK) was deleted in *Mycobacterium tuberculosis*, it resulted in the disruption of cell viability, further establishing the attractiveness of targeting the SP in general, and SK in particular [17].

Due to significant costs, slow progress, and high rates of failure in traditional drug discovery, repurposing existing drugs for the treatment of various diseases, both common and rare, is gaining popularity. This approach utilizes already approved and de-risked compounds, potentially reducing overall expenses and shortening development duration [18]. In the present study, we utilized computer-aided drug design (CADD) approaches, namely molecular docking, Molecular Mechanics-Generalized Born Surface Area (MM-GBSA) calculations, molecular dynamics (MD) simulations, and Density Functional Theory (DFT) calculations, aiming to investigate the repurposing possibility of US FDA-approved drugs to be used as *H. pylori* shikimate kinase (HpSK) inhibitors.

## 2. Results and Discussion

### 2.1. Molecular Docking and MM-GBSA Calculations

The crystal structure of *H. Pylori* shikimate kinase in its unbound conformation was used for the docking studies (PDB: 1ZUH). As the selected crystal structure of *H. Pylori* shikimate kinase lacked a co-crystallized ligand, it was crucial to employ a reliable and effective approach to identify the enzyme’s active pocket where the inhibitor would bind. The active site was predicted with SiteMap and was validated by comparing it with a *H. Pylori* shikimate kinase crystal structure that was bound to a shikimate ligand (PDB: 3MUF). The SiteMap-predicted active site was found to match the shikimate-bound active site; thereby, the active site for the studied protein was the competitive orthosteric site in its wild unbound conformation. Molecular docking occupies an essential place in the process of computational drug discovery. This stems from the fact that it is used to examine the interactions between a target molecule and a ligand through both bonding and non-bonding interactions. Despite its limitation in neglecting the flexibility of biomolecules, it remains a reliable approach for generating preliminary insights into molecular interactions [19,20]. In the Glide module of docking, the HTVS method was applied to screen a total of 1615 US-FDA-approved agents following their preparation. Subsequently, the top 111 candidates from the initial pool underwent extra-precision (XP) docking. The XP docking mode is known for its superior accuracy and precision compared to other modes, albeit at a higher computational cost. This mode was utilized for initial compound screening and further refinement based on XP docking scores. Glide’s scoring function integrates empirical and force-field-based parameters to calculate binding energy, allowing for the identification of the most favorable docking poses [3,4]. As a reference control, a shikimate molecule was prepared as well and docked against the target protein, as the candidate drugs were to be tested as competitive inhibitors of shikimate kinase. The docking score of shikimate was −5.867 kcal/mol, and all the of other molecules were compared against it. Worthy of note is that there were 31 compounds that presented docking scores that were better than that of the reference ligand. To account for additional critical factors influencing molecular binding beyond molecular docking, MM-GBSA calculations were conducted on the chosen 31 compounds, along with shikimate.

**Figure 2:**
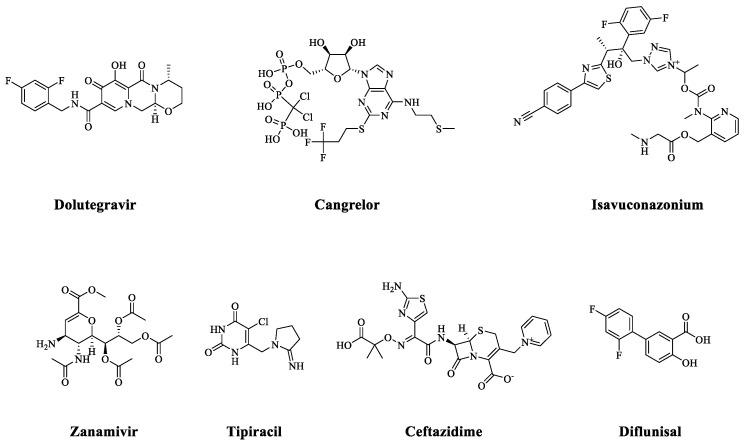
The chemical structures of the seven hits.

The MM-GBSA analysis enhances the accuracy of binding energy calculations compared to molecular docking energies as it follows a more theoretically robust approach rather than relying on empirical scoring functions commonly used in molecular docking [21]. The 31 compounds exhibited binding free energy (dG bind energy) values ranging from 3.61 to −55.92 kcal/mol, while shikimate showed a dG bind energy of −34.24 kcal/mol. Among the 31 compounds, 2 compounds had dG bind energies higher than −10 kcal/mol, 12 compounds fell between −10 and −30 kcal/mol, and 17 compounds had values more than −30 kcal/mol (Table S1). These seventeen compounds were further refined based on their identity and indication of use, resulting in the identification of seven promising candidates, detailed in Table 1, and their chemical structures, shown in Figure 2.

In a subsequent filtration process, three drugs—Dolutegravir, Cangrelor, and Isavuconazonium—were selected for further analysis. These compounds were prioritized based on their drug nature, favorable clinical safety profiles, and superior MM-GBSA binding free energy values compared to the reference ligand. Dolutegravir, known for its use in antiretroviral (HIV-1) therapy, has a well-documented clinical safety profile and excellent pharmacokinetics [22]. Meanwhile, Cangrelor is the sole intravenous P2Y12 receptor inhibitor currently available, known for its strong, consistent, and quickly reversible anti-platelet activity [23]. The third option, Isavuconazonium, is a well-established antifungal prodrug recognized for its demonstrated safety and effectiveness [24].

The ligand interaction diagram generated by Maestro revealed distinct interaction patterns for the control inhibitor, shikimate, and the docked compounds (Dlutegravir, Cangrelor, and Isavuconazonium) with the active binding site residues of the target protein. These differences in binding interactions provide insights into the structural features influencing ligand affinity and specificity.

Shikimate, serving as the control ligand, demonstrated robust hydrogen bonding with key amino acid residues at the active binding site. It formed hydrogen bonds with Gly11 (1.90 Å), Ser15n (1.73 Å), Asp31 (2.34 Å), and Asp33 (1.94 Å). These interactions suggest a strong stabilizing effect of shikimate within the binding pocket, likely contributing to its inhibitory efficacy.

**Figure 3:**
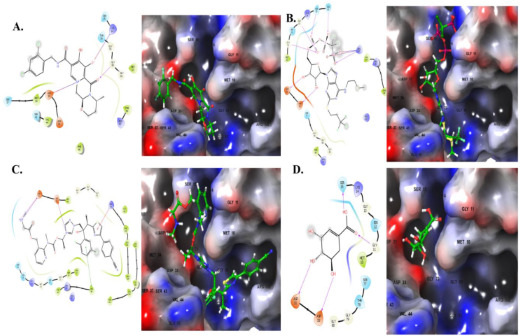
The 2D and 3D diagrams of the top compounds and shikimate with the HpSK (PDB ID: 1ZUH). (**A**) Dlutegravir, (**B**) Cangrelor, (**C**) Isavuconazonium, (**D**) shikimate.

As is shown in Figure 3, Dolutegravir exhibited a distinct binding profile characterized by hydrogen bonding with Gly80 (1.87 Å) and salt bridges with Lys14 and Asp33. These interactions imply a combination of polar and electrostatic contributions to its binding affinity.

Cangrelor displayed an extensive interaction network within the active site. It formed hydrogen bonds with Gly11 (2.12 Å), Gly13 (1.88 Å), Ser15 (1.72 Å), Ser16 (2.52 Å), Asp31 (2.23 Å), Asp33 (2.55 Å), and Arg107 (2.17 Å). Additionally, two salt bridges with Arg107 were observed, emphasizing the strong electrostatic stabilization provided by this compound.

Isavuconazonium exhibited a unique interaction profile, including salt bridges with Met10, Asp31, and Asp33. It also formed π–π stacking interactions with Phe48 and a π–cation interaction with Arg132. These non-covalent interactions, particularly the π–π stacking and π–cation interactions, likely enhance the stabilization of Isavuconazonium within the binding site.

Comparatively, shikimate’s interaction profile was dominated by hydrogen bonds, contributing to its stable binding. Dlutegravir showed fewer hydrogen bonds but incorporated salt bridges, reflecting a different binding strategy. Cangrelor stood out with the highest number of hydrogen bonds and additional salt bridges, suggesting a potentially stronger and more specific binding affinity. In contrast, Isavuconazonium leveraged a combination of salt bridges, π–π stacking, and π–cation interactions, which may provide unique advantages in terms of binding stabilization and target selectivity. These findings highlight the distinct binding mechanisms of the tested compounds compared to the control inhibitor and underscore the potential of Dlutegravir, Cangrelor, and Isavuconazonium as promising candidates for further development. This goal can be achieved via testing the stability of those compounds in molecular dynamics simulation studies.

### 2.2. Molecular Dynamics (MD) Simulation

**Figure 4:**
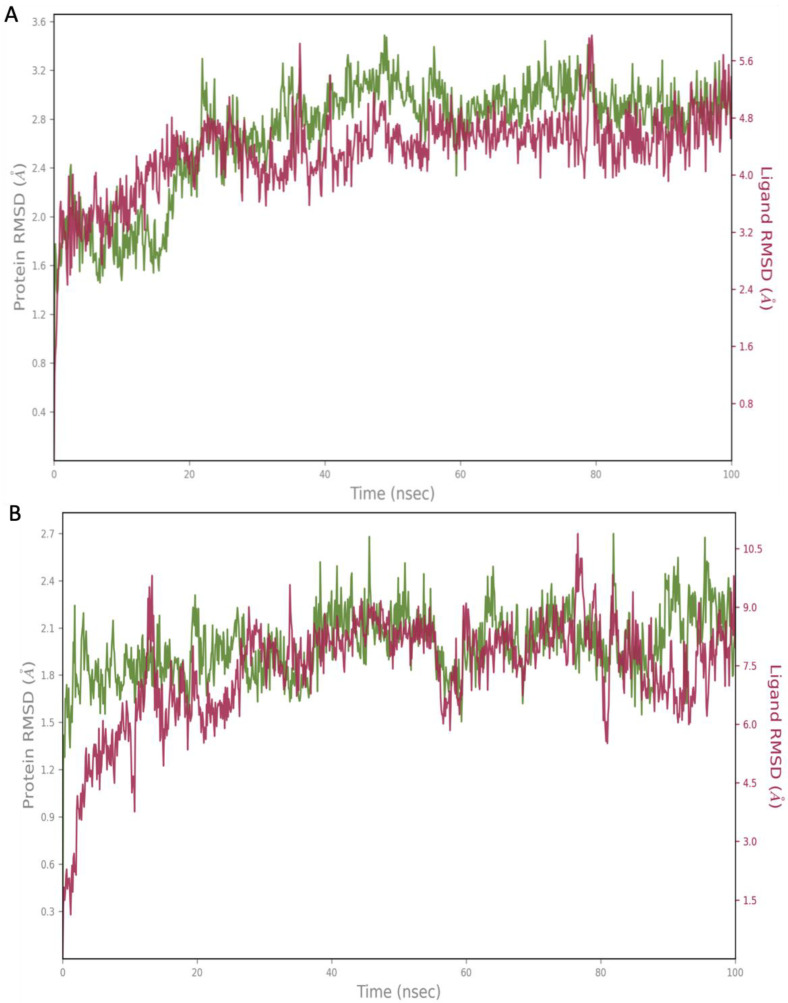
The root mean square deviation (RMSD) graphs for the hit compounds (**A**) Cangrelor and (**B**) Isavuconazonium. The green graph shows the fluctuations in the protein backbone from the initial reference point, while the red shows the ligand fluctuations. The RMSD profile of the ligand with respect to its initial fit to the protein binding pocket indicates that all ligands did not fluctuate beyond a 2–5 Å range.

The hit compounds Cangrelor and Isavuconazonium were chosen for further analysis by MD perturbation. Molecular dynamic simulation studies were considered essential in order to verify the strength of the binding interactions. Cangrelor and Isavuconazonium both showed a robust binding profile within the HpSK protein binding pocket. Root mean square deviation (RMSD) plot analysis was used to measure the average displacement of atoms of a particular frame with respect to a reference frame. The analysis revealed a stable binding profile, as indicated by an average RMSD fluctuation range within 4 Å of the ligand with reference to the protein backbone throughout the simulation period. The RMSD plots for the top two compounds selected for MD studies are shown in Figure 4. These candidates were selected based on the favorable nature of the binding interactions formed with key residues of the target protein. Cangrelor and Isavuconazonium also represent good candidates for drug repurposing studies. Figure 4 reveals that after an initial short period of stabilization, the RMSD fluctuations remain within a narrow 2 Å range, highlighting the conformational stability of the ligand (red) relative to the protein backbone (green).

**Figure 5:**
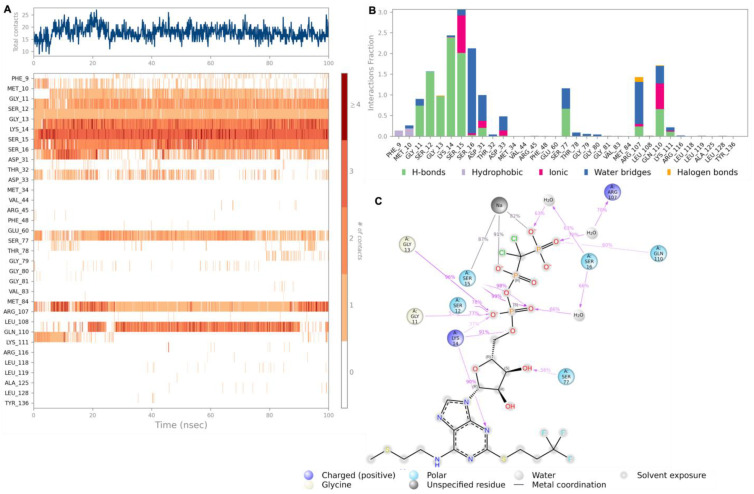
The interaction diagrams of hit compound Cangrelor with the HpSK binding pocket. (**A**) The interaction of HpSK residues with Cangrelor in each trajectory frame. The depth of color indicates a higher interaction with the contact residues. (**B**) The protein–ligand contacts showing the bonding interactions fraction and the nature of these interactions. (**C**) A graphical 2D illustration of compound 3 interacting with the protein residues during MD simulation.

A strong and robust binding interaction profile is observed for Cangrelor as shown in Figure 5. Cangrelor was found to interact with a number of key residues in the HpSK binding pocket including Lys 14 and Ser 15, forming stable hydrogen bonds for over 90% and 80% of the simulation period, respectively. Additionally, a stable hydrogen bond interaction was observed between the ligand and residue Gly 13 for 96% of the simulation period. The key interactions and the strongest ligand interactions with specific residues throughout the simulation period are depicted in Figure 5A,B. Figure 5C highlights all significant interactions displayed by the ligand and interacting residues occurring for over 30% of the simulation period. Cangrelor is an FDA-approved anti-platelet medication prescribed for the prevention of myocardial infarction; it acts by inhibiting ADP-mediated platelet aggregation through the selective and reversible inhibition of the P2Y12 receptor. The clinical accessibility and safety profile of this medication renders it exceptionally amenable to subsequent repurposing studies.

**Figure 6:**
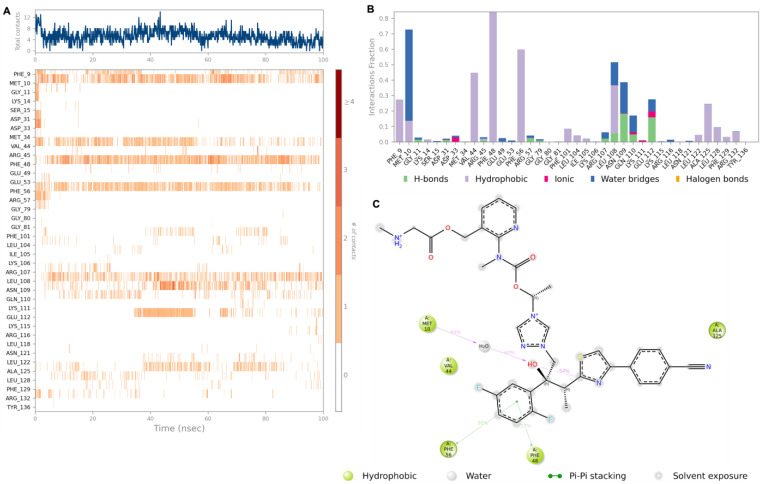
The interaction diagrams of hit compound Isavuconazonium with the HpSK binding pocket. (**A**) The interaction of HpSK residues with Isavuconazonium in each trajectory frame. The depth of color indicates a higher interaction with the contact residues. (**B**) The protein–ligand contacts showing the bonding interactions fraction and the nature of these interactions. (**C**) A graphical 2D illustration of compound 3 interacting with the protein residues during MD simulation.

Figure 6 shows the binding profile of hit compound Isavuconazonium with residues in the HpSK binding pocket. Isavuconazonium forms strong and moderate interactions with the key binding residues Met 10, Phe 56, and Phe 48 of the receptor binding pocket, hydrophobic and water-bridge interactions with Met 10, and hydrophobic interactions with both Phe 56 and Phe. The complex hydrophobic and water-bridge interactions observed between Isavuconazonium and the HpSK residue Met 10 were observed for over 40% of the simulation period. Isavuconazonium is a potent antifungal medication which has been approved by the FDA for the treatment of invasive aspergillosis. Like Cangrelor, its approval status and safety profile provide an attractive foundation to launch the development of subsequent precursors that can be modified towards selective HpSK inhibition.

### 2.3. Density Functional Theory (DFT) Calculations

**Figure 7:**
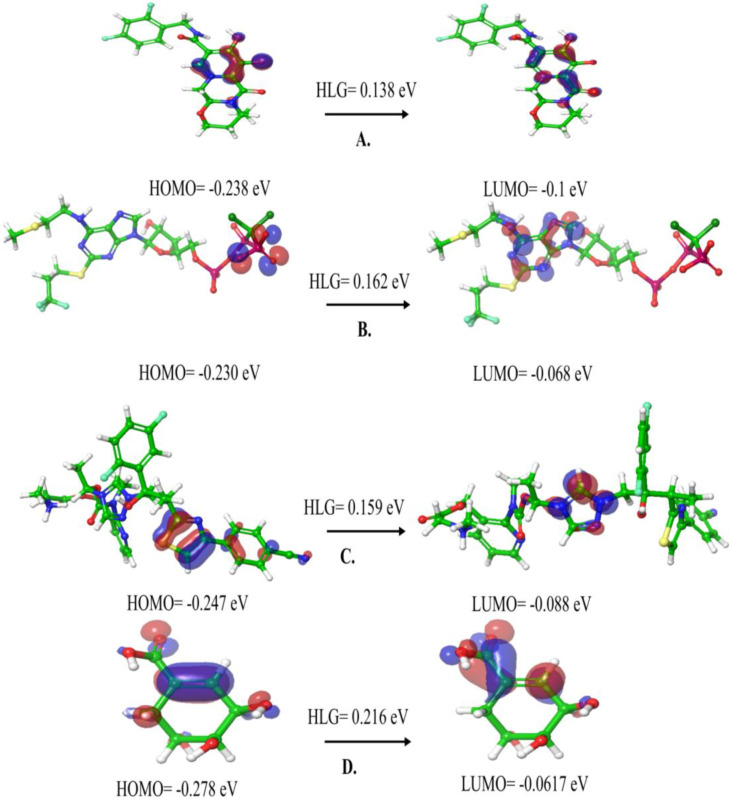
HOMO and LUMO plots of the top three compounds and shikimate. (**A**) Dlutegravir, (**B**) Cangrelor, (**C**) Isavuconazonium, (**D**) shikimate.

DFT serves as a powerful approach for assessing the energy levels of orbitals within compounds, enabling the anticipation of ligand stability and charge transfers at the interaction site. This method proves invaluable in computing the electronic structure of a material, providing beneficial insights into the behavior and reactivity of chemical compounds [25]. The electronic structure of the three selected compounds in addition to shikimate was calculated; High Occupied Molecular Orbitals (HOMOs), Low Unoccupied Molecular Orbitals (LUMOs), and the energy gap (HLG) between them were determined and presented in Figure 7.

For Dlutegravir (Figure 7A), the HOMO and LUMO were located on the (3S,7R)-13-carbamoyl-12-hydroxy-7-methyl-9-oxo-4-oxa-5,8-diazatricyclo[8.4.0.0^{3,8}]tetradeca-1(14),10,12-trien-1-ylium-11-olate moiety with energies of −0.238 and −0.1 eV, respectively. The docking results revealed that this moiety interacted with the residues LYS14, ASP33, and GLY80 of HpSK.

Regarding Cangrelor (Figure 7B), the HOMO relies on the peripheral phosphonate group, with energy of −0.230 eV, while the LUMO relies on the purine-6-amine moiety with an energy of −0.068 eV. According to the molecular docking, the phosphonate group interacted with ARG107, while the purine-6-amine moiety showed no interaction.

Lastly, for Isavuconazonium (Figure 7C), its HOMO was situated on the 4-(1,3-thiazol-4-yl)benzonitrile moiety with an energy of −0.247 eV. The LUMO was situated on 1,2,4-triazol moiety with an energy of −0.088 eV. These two moieties interacted with the ASP33 and ARG132 of the HpSK protein, respectively.

On the other hand, regarding the shikimate (Figure 7D), the HOMO and LUMO were disseminated throughout the small molecule with energies of −0.278 and −0.0617 eV, respectively. Shikimate exhibited interactions with the GLY11, SER15, ASP31, and ASP33 of HpSK.

When the HOMO value is high, it signifies the compound’s strong electron-donating capability, and conversely, a low HOMO value indicates the opposite [26]. For instance, Cangrelor exhibits superior electron donation potential with an energy level of −0.230 eV, whereas shikimate shows weaker electron donation ability at −0.278 eV. Furthermore, a narrow energy gap (HLG) between the HOMO and LUMO implies smoother electron transitions, translating to increased reactivity, improved photo-stability, and ultimately enhancing drug stability. The compounds can be ranked in the following order: shikimate (0.216 eV) > Cangrelor (0.162 eV) > Isavuconazonium (0.159 eV) > Dlutegravir (0.138 eV). All the three drug candidates have HLG values less than the shikimate, which means they are more reactive and facilitate electronic transition more than shikimate.

## 3. Materials and Methods

### 3.1. Software

In this study, all the in silico analyses were conducted using Maestro version 2024-4 (procured via the RDIA Grant 12990-iau-2023-iau-R-3-1-HW: P.O. 6947). The Maestro platform is part of the Schrödinger software suite [27]. The desktop workstation was equipped with an Intel^®^ Core™ i7-10700F Processor, Linux Ubuntu 22.10 operating system, and an RTX 5000 graphics card.

### 3.2. Preparation of Ligands

A total number of 1615 US-FDA-approved agents listed on the ZINC database were subjected to preparation. The LigPrep tool in the Maestro 12.8 software suite was utilized with default settings to process the collected ligands, aiming to minimize potential computational errors. This procedure yielded energy-minimized and structurally optimized 3D chemical assemblies. Structural inconsistencies within the ligand library were corrected, and plausible ionization states were generated under physiological pH conditions (7 ± 2). Then, the MacroModel module of Maestro was exploited for the purpose of preparing shikimate (the control ligand) for the consequent processes of molecular docking. In the MacroModel module, this goal was achieved by refining both the geometry and conformational flexibility of the ligands before molecular docking [28,29]. This refinement involved energy minimization, where bond lengths, angles, and torsions were optimized to reach stable, low-energy states, as well as conformational sampling to explore different ligand shapes and identify potential binding configurations. Utilizing the OPLS force-field, MacroModel effectively captured the physical and chemical characteristics of shikimate, ensuring its stability and suitability for the docking process.

### 3.3. Protein Target Retrieval and Preparation

From the protein data bank that is available at https://www.rcsb.org (accessed on 21 March 2024) the Apo-form crystal structure of Helicobacter pylori shikimate kinase (HpSK) enzyme was brought to the Maestro interface due to its proper quality (PDB ID: 1ZUH) [30]. The Apo-form was chosen for virtual screening to avoid the limitations on inhibitor diversity that may have arisen from using the closed-form structure induced by bound ligands. The quality features found in this target protein were its high-resolution structure (1.80 Å) and its X-ray crystallographic source. These metrics provided a clear visualization of the overall protein structure, backbone, and side-chain conformations. In order for the protein to be ready for any handling, it had to be subjected to a thorough process of preparation to correct the flaws in the protein data bank crystal structure. Usually, the imperfections in the structure occur as an improper bond order and missing side chains. Using the Schrödinger suite’s Protein Preparation Wizard, multiple corrections to the protein under study were made [31]. These adjustments included the following: detecting disulfide bonds, adding hydrogen atoms, assigning correct bond ordering, and fixing mislabeled components. In the end, this preparation method uncovered and fixed structural problems to guarantee a solid basis for further docking. This was assured by making a protein reliability report [32].

### 3.4. Site Mapping and Generation of Receptor Gride

The SiteMap module in Maestro was used to predict the crystal shikimate kinase active site [30]. In order to choose targetable sites, SiteMap provides an extensive set of tools for identifying, analyzing, and visualizing possible binding sites. This is achieved via evaluating factors such as shape, size, and hydrophobic or hydrophilic properties and ranking all the possible pockets by means of a site score [33,34]. After this, the coordinates of the pocket with the highest site score were used for receptor grid generation, ensuring the precise determination of the SK active site.

### 3.5. Molecular Docking

For the molecular docking analysis, the Glide module was used. This module enabled precise and reliable interactions between the ligand and target protein. High Throughput Virtual Screening (HTVS), SP, and extra-precision (XP) are the three docking methods available in the Glide module [35]. To varied degrees, each approach strikes a balance between speed, accuracy, and scoring functions. The XP mode uses a stricter scoring process with improved sampling, although HTVS and SP use similar scoring mechanisms. In particular, chemicals that are less compatible with the protein’s binding site are penalized by the XP approach [36,37,38,39]. For these variable ranges of accuracy and precision, the three modes of docking were performed to assess the binding affinities of the ligands to the shikimate kinase binding site in a consequent manner. To be exact, the approved drugs, processed during ligand preparation, were docked into the active shikimate binding site using the HTVS mode. Molecules that achieved high docking scores in HTVS were subsequently docked using the SP mode, followed by an additional docking in the XP mode for refined accuracy.

### 3.6. Free Binding Energy Calculation (MM/GBSA)

After completing the docking procedure, the ligands with the highest docking scores for both proteins were selected. The docking poses of the approved drugs in the shikimate kinase binding pocket were then used to compute the free binding energy using the MM/GBSA method, implemented in the Prime module with its default settings [40]. The binding energy was calculated using the following formula:

E = E_complex_ − E_protein_ − E_ligand_, (1)

where E_complex_ represents the energy of the protein-ligand complex, E_protein_ denotes the energy of the protein, and E_ligand_ refers to the energy of the ligand.

### 3.7. Molecular Dynamic (MD) Simulations

Molecular dynamic (MD) simulation investigations were performed using Desmond, a component of the Schrödinger’s Maestro drug discovery computational platform, as previously described [41]. Briefly, the protein complex with the hit compound docked in its optimal conformation was first minimized using the Protein Preparation Wizard. The protein-hit complex was then prepared for molecular dynamic simulation using the system builder application. A suitable simulation environment was engineered using the default settings and a TIP3P water-based solvent system. An orthorhombic simulation box was generated with a buffer parameter at a distance of 10 Å from the protein surface. To attain isosmotic conditions, 0.15 M of sodium chloride was added and the system was neutralized through the addition of a required number of counter ions. A temperature of 300 K and an atmospheric pressure of 1.013 bar were used to carry out MD simulation calculations. The duration of the simulation period was set as 100 nanoseconds and a total of 1001 frames were saved. The resulting trajectory was analyzed and the simulation analysis and interaction diagram tool were used to present the results simulation.

### 3.8. Density Functional Theory (DFT) Calculations

In order to determine the molecular reactivity of the successfully docked compounds, DFT calculations were performed. DFT is an effective method for evaluating the energy levels of orbitals in compounds, allowing for further prediction of ligand stability and charge transfer at the interaction site. Technically, DFT calculations were conducted using the Jaguar tool within the Maestro platform. The B3LYP method, paired with a 6–31G+* basis set, was chosen along with the Poisson–Boltzmann Finite (PBF) solvent model [42]. This analysis involved calculating the values of the HOMO (Highest Occupied Molecular Orbital) and LUMO (Lowest Unoccupied Molecular Orbital) together with the HLG (HOMO-LUMO Gap).

## 4. Conclusions

This study successfully employed different computational experiments to investigate the repurposing potential of FDA-approved drugs as inhibitors of *Helicobacter pylori* shikimate kinase (HpSK). By screening 1615 FDA-approved drugs through molecular docking and subsequent extra-precision docking, followed by MM-GBSA calculations, three compounds—Dolutegravir, Cangrelor, and Isavuconazonium—were identified as promising candidates based on their superior binding free energies, favorable drug properties, and safety profiles. Robust and stable binding profiles for both Isavuconazonium and Cangrelor were verified via molecular dynamics simulation. Additionally, Density Functional Theory (DFT) revealed that these compounds exhibited lower energy gaps (HLGs) than the reference ligand, shikimate, indicating enhanced reactivity and more efficient electronic transitions. The results indicated that the identified drugs, while not optimal for repurposing, may serve as potential candidates for experimental evaluation against *Helicobacter pylori* shikimate kinase. The findings also indicated that diflunisal is a promising candidate for therapeutic repurposing and warrants further investigation.

## Figures and Tables

**Table 1:** The seven top-ranked compounds according to their docking score, identity, and MM-GBSA dG bind energy on the HpSK active site.

No. *	ZINC_ID	Medication	Docking Score (XP)	MM-GBSA dG Bind **
1	ZINC000058581064	Dolutegravir	−7.947	−35.05
2	ZINC000003918138	Zanamivir	−7.727	−32.43
3	ZINC000085537017	Cangrelor	−7.645	−35.73
4	ZINC000100032379	Tipiracil	−7.285	−31.56
5	ZINC000003871960	Ceftazidime	−6.120	−33.03
6	ZINC000029571072	Isavuconazonium	−6.069	−53.07
7	ZINC000000020243	Diflunisal	−5.981	−33.35
8	Shikimate	-	−5.867	−34.24

* hits are arranged according to their XP score. ** calculated in kj/mol.

## Data Availability

In silico drug experiments using molecular docking to target HpSK receptor (PDB ID: 1ZUH) were obtained from the Research Collaboratory for Structural Bioinformatics (RCSB) Protein Data Bank (PDB). PDB DOI: https://doi.org/10.2210/pdb1ZUH/pdb. The FDA-approved drugs’ structures were downloaded from the ZINC database: https://zinc.docking.org/substances/subsets/fda/ (accessed on 21 January 2025). All data generated or analyzed during this study are included in this published article.

## Supplementary Materials

The following supporting information can be downloaded at: https://www.mdpi.com/article/10.3390/ph18020174/s1, Table S1: Docking scores and binding free energy (dG bind) values for the hit compounds and the control compound (Shikimate) on the HpSK active site.
