# Supplementary material for: Repurposing FDA-Approved Agents to Develop a Prototype Helicobacter pylori Shikimate Kinase (HPSK) Inhibitor: A Computational Approach Using Virtual Screening, MM-GBSA Calculations, MD Simulations, and DFT Analysis
Source: Pharmaceuticals (Basel). 2025 Jan 27;18(2):174. doi: 10.3390/ph18020174 (PMC11858459; doi:10.3390/ph18020174)
Supplement: Supplementary file 1 [file pharmaceuticals-18-00174-s001.zip › pharmaceuticals-3415959-supplementary.pdf]

Table S1: Docking scores and binding free energy (dG bind) values for the hit compounds and the control compound (Shikimate) on the HpSK active site.

No.	ZINC_ID	Docking score	MMGBSA dG Bind (kcal/mol)
1.	ZINC000003830946	-8.244	-35.79
2.	ZINC000000085733	-8.208	-2.15
3.	ZINC000008035395	-8.009	-54.18
4.	ZINC000058581064	-7.947	-35.05
5.	ZINC000003782550	-7.755	-30.62
6.	ZINC000003918138	-7.727	-32.43
7.	ZINC000085537017	-7.645	-35.73
8.	ZINC000000901736	-7.540	-11.58
9.	ZINC000000000061	-7.366	-20.02
10.	ZINC000100032379	-7.285	-31.56
11.	ZINC000008143864	-7.055	-45.01
12.	ZINC000000004949	-6.954	-17.92
13.	ZINC000053022902	-6.916	-29.66
14.	ZINC000001531008	-6.753	-31.53
15.	ZINC000003927870	-6.710	-25.54
16.	ZINC000003920355	-6.604	-45.58
17.	ZINC000008214418	-6.360	-48.77
18.	ZINC000100071256	-6.313	-20.18
19.	ZINC000001540998	-6.235	-31.94
20.	ZINC000003801919	-6.207	3.61
21.	ZINC000001533877	-6.121	-23.40
22.	ZINC000003871960	-6.120	-33.03
23.	ZINC000029571072	-6.069	-53.07
24.	ZINC000008220909	-6.067	-43.25
25.	ZINC000164528615	-6.038	-55.92
26.	ZINC000003830813	-6.021	-16.97
27.	ZINC000000020243	-5.981	-33.35
28.	ZINC000001533877	-5.961	-25.06
29.	ZINC000003809490	-5.915	-12.84
30.	ZINC000003830813	-5.914	-24.17
31.	ZINC000003830813	-5.881	-16.91
32.	Shikimate	-5.867	-34.24
